# Comparative analysis of the physical and phenotypic traits of native cattle (*Bos indicus*) in the Tarai region of North Bihar for conservation

**DOI:** 10.14202/vetworld.2025.95-101

**Published:** 2025-01-14

**Authors:** Krishna Mohan, Pramod Kumar, Anandamoy Kundu

**Affiliations:** Centre of Excellence on Indigenous Breed, Dr. Rajendra Prasad Central Agricultural University, Pusa, Samastipur, Bihar, India

**Keywords:** body weight, conservation, phenotypic traits, physical, Tarai cattle

## Abstract

**Background and Aim:**

The evaluation of the phenotypic and morphological characteristics of indigenous breeds may help to frame breeding policies and plans to implement breed conservation and improvement programs to increase the efficiency of the native breed of the Tarai region. This study aimed to determine the phenotypic and morphological characteristics of indigenous cattle of the Tarai region of North Bihar.

**Materials and Methods:**

A field study was undertaken in 32 villages belonging to 13 blocks of the East and West Champaran districts of Bihar. Animals (n = 562) of different age groups were studied to evaluate their phenotypic and morphological characteristics.

**Results:**

It revealed that cattle of the Tarai region are small breeds, and the heights at withers in the females and males were 104.2 ± 0.32 cm and 115 ± 0.27, respectively, with a significant (p = 0.000) difference between sexes. In addition, heart girth was significantly (p = 0.000) higher in males than females. Body length and chest girth were 101.6 ± 0.13 cm and 132.6 ± 0.25 in adult females and 114.8 ± 0.23 cm and 145.7 ± 0.15 cm in adult males, respectively. Body weight also differed significantly (p = 0.001), with 180.9 ± 1.12 and 208.7 ± 1.91 cm for females and males, respectively. Among the draught animals of India, the region cows were comparatively low milkers, with an average lactation yield of 680.2 ± 4.52 kg with a mean lactation length of 224.5 ± 2.06 days and a peak yield of 3.4 ± 0.06 kg/day. The age at first calving and calving interval of Tarai cattle was recorded 32.4 ± 0.22 months and 16.2 ± 0.12 months, respectively.

**Conclusion:**

The results of this study could serve as a potential guide for the establishment and identification of new cattle breeds based on the phenotypic and morphometric characteristics of the cattle in Tarai region of North Bihar using baseline data. The data generated from this study can be useful for new Tarai breed identification and serve to establish long-term selective breeding programs for Tarai cattle in the region.

## INTRODUCTION

The indigenous breeds of cattle in Bihar have unique characteristics that help them to be well-adapted to the tropical climate of Bihar. The economy of North Bihar largely depends on agriculture and related sectors, and these indigenous cattle breeds from the Tarai region are well adapted to high temperatures during summer, humidity, and rainfall. These indigenous cattle play a crucial role in converting agricultural by-products into efficient draught power to a greater extent and alleviating the livelihood status of livestock farmers using milk and dung to a lesser extent. However, in addition to other farm operations such as draught power, dung from these Tarai cattle is widely used for fueling farmers.

The indigenous breed of farm animals has the capacity to adapt to harsh climatic conditions with minimal management intervention in terms of feed, fodder, and health care. These breeds are also better adapted to withstand tropical diseases and heat tolerance [1]. The indigenous breeds of cattle result from gradual evolutionary processes and are integral to agriculture. Due to the unplanned breeding and introduction of exotic germplasm through crossbreeding, indigenous breeds face genetic degradation and dilution of the purity of the breed. However, sufficient phenotypic and morphometric information is essential for selecting superior animals for conservation and breeding to produce more productive herds [2]. Therefore, the conservation of indigenous breeds is essential for long-term survival, particularly considering climate change.

Measurements of body conformation are an important part of an animal’s phenotypic characterization, and this can be determined by individual animal conformation and morphometric characteristics. A combination of multiple morphometric measurements can provide a superior guide for describing the type and function of domestic animals [3]. Qualitative traits are as important as quantitative traits for animal identification. Coat color type, navel flap, and tail length (TL) are important categorical characteristics with larger association values used for breed identification [4].

The evaluation of indigenous breed phenotypic and morphological characteristics under field conditions may help frame breeding policies and plans to take up breed conservation and improvement programs to increase the efficiency of the indigenous breed in the area of the study. Nevertheless, no research has been done to investigate the possibilities of identifying the distinctiveness in phenotypic and morphological traits of native cattle, particularly in the border region of Indo-Nepal and the Tarai region of North Bihar.

The phenotypic traits, practical utility, and adaptability of cow herds raised and acclimated to India’s diverse agroclimatic environments and production systems exhibit remarkable variation [5]. The centuries-long process of domestication, mutation, selective breeding, environmental adaptability, and genetic drift are all responsible for the genetic variety found in cattle breeds [6]. The preservation of native breeds is necessary for scientific research, future genetic insurance, cultural and ethical requirements, and environmental support. It is important to take precautions against future losses of genetic variety in dairy cattle herds. In addition, local breed utilization, conservation, and genetic improvement should be combined [7].

To provide genetic insurance for future scientific research, native breeds must be preserved.

The preservation of native breeds is necessary for scientific research, future genetic insurance, ecological health, cultural and ethical requirements, and energy sources. The native cow breeds are well suited to the tropical climate because they have a “thermometer gene” and a unique genetic variant in the *HSP70* gene family, among other distinctive traits [1]. The indigenous breeds of cows have gained popularity recently [8], and their milk carries the A2 allele, which is considered beneficial for human health. Numerous breeds are currently susceptible to rapid genetic degradation and dilution due to haphazard breeding and the introduction of exotic material through crossbreeding [9]. Effective management of indigenous cattle resources includes identification, characterization, evaluation, documentation, and conservation.

Hence, the present investigation was undertaken to study the phenotypic and morphological characteristics of Tarai indigenous cattle and to record the unique features of the production and reproduction performance of the indigenous breed in the breeding tract of the Tarai region of North Bihar. However, one of the recognized indigenous breeds of cattle in North Bihar is Bachaur, and the breed is distributed throughout the Gangetic plains of North Bihar.

## MATERIALS AND METHODS

### Ethical approval

This study did not require specific ethical approval as per the institutional guidelines. However, the survey was carried out in accordance with the animal welfare guidelines laid by the Institutional Animal Ethics Committee. The study was conducted using adequate measures to minimize pain or discomfort to animals during survey work.

### Study period and location

This study was conducted from August 2021 to November 2022. This study was conducted in two districts, East Champaran and West Champaran, including the Tarai region and border areas of Indo-Nepal in north Bihar. The extension of the Shiwalik ranges and its Tarai region can be found in the north-western part of Western Champaran in Bihar. Bihar is situated between 24° 20’ 10” North and 27° 31’ 15” North latitudes and 83° 19’ 50’’ East and 88° 17’ 40’’ East longitudes. It is 483 Km long (East to West) and 345 Km wide (North to South). Bihar is situated between humid West Bengal (West) and sub-humid Uttar Pradesh (East). Hence, Bihar has a transitional climate between humid and sub-humid.

### Survey and field studies

**Figure 1:**
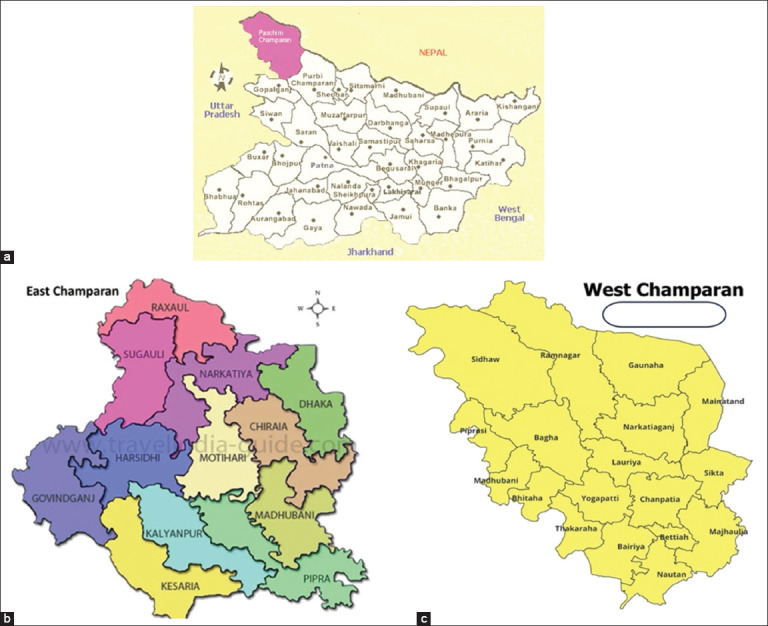
(a) Map of Bihar state (Source: http://www.mapsofindia.com/). Map of study areas of (b) East and (c) West Champaran districts of North Bihar [Source: National Informatics Center].

Overall, 32 villages belonging to 13 blocks of the East and West Champaran districts of Bihar were selected to study the phenotypic and morphological traits of the indigenous cattle of the Tarai region. Visits were made to different villages in east and west Champaran, including the Tarai region and border area of the Indo-Nepal region of North Bihar, to collect information on cattle in this region (Figure 1). Information on morphological traits [body length (BL), wither height (WH), chest girth, horn length (HL), face width (FW), ear length (EL), TL, and body weight (BW)] was collected. Animals (n = 562) of both sexes (n = 217 males; n = 345 females) of cattle belonging to different age groups were studied.

The study was conducted in this region due to low milk production and poor performance of animals for dairy purposes, and these two districts, namely, East Champaran and West Champaran from North Bihar, were selected on the basis of stratified random sampling. Physical and morphological traits were measured; however, information on lactation and reproductive performance was obtained through interacting with cattle owners on a pre-designed questionnaire developed by National Bureau of Animal Genetic Resources, Karnal with slight modification recommended for this purpose.

### Housing and feeding management in the Tarai region of north Bihar

The farmers and livestock keepers in the Tarai region of North Bihar were either landless or had little land. Despite the poor economic conditions, the majority of livestock farmers in the Tarai area were provided housing and shelter for their animals, but the shelters lacked basic amenities and had poor ventilation. These Tarai cattle were generally housed outside in the shade during the day and within sheds at night.

The sheds were mostly *kachcha* type with walls made of bricks and mud, and the roof was either made of thatched, paddy straw, or asbestos. The climatic conditions in this area forced farmers to provide at least the minimum requirement of housing, especially in the winter when the minimum temperature in the area reaches around 6°C–7°C.

The livestock farmers fed their animals twice a day, and only a few of them fed 3 times a day. Furthermore, most livestock farmers fed wheat straw (*Bhusa*) as dry fodder, and only a few fed rice straw or both (wheat straw and rice straw) to their animals. Most Tarai cattle were stall-fed by farmers, and some allowed their cattle to graze apart from stall feeding. The different management practices adopted by the farmers for Tarai cattle were studied using observation and questionnaires. The phenotypic and morphological traits of the Tarai cattle were recorded as per the standard procedure, and the reproductive traits were determined with the help of a questionnaire. The data related to feeding and housing management practices in this region were also collected during the survey.

### Statistical analysis

Morphological data were statistically analyzed and expressed as percentages, means, standard deviation (SD), and standard error (SE) using Microsoft Excel 2016, version 16.0.4324.1002 (Microsoft Corporation, USA). The BWs of the breed across various ages were calculated using a modified version of Shaeffer’s formula, which included an adjustment to convert pounds to kilograms, as outlined below:

BW (kg) = (Chest girth in inches)^2^ × (BL in inches)/300 BW (kg) × 0.4536

## RESULTS

### Phenotypic and morphological measurement

The different phenotypic and morphological measurements of the Tarai cattle under field conditions during the survey are given in Tables 1 and 2. All morphometric values of wither height (WH), BL, heart girth (HG), and BW were significantly (p < 0.01) greater in males than in females, except for horn circumference (HC), FW, and TL.

**Table 1:** Description of the morphological measurements of the Tarai cattle of North Bihar.

Body measurement (cm)	Measurement
HL	Distance from nape to the rostral end of the muzzle.
FW	Distance of the widest head points.
EL	Distance from root to ear end point.
BL	Horizontal distance from the point of shoulder to pin bone.
HG	Place the measuring tape around the animal at the point of the smallest circumference, just behind the forelegs.
WH	Distance (vertical) from the bottom of the front foot to the highest point above wither.
TL	Distance from the base of the tail proximal end of the first coccygeal bone to the distal end of the last coccygeal bone.
LH	Distance from the base of horn to the tip.

HL=Head length, FW=Face width, EL=Ear length, BL=Body length, HG=Heart girth, WH=Wither height, TL=Tail length, LH=Horn length

**Table 2:** Mean ± SE for phenotypic and morphological characteristics of the Tarai cattle of North Bihar.

Traits	Calves and Young stocks (<1–3 years)	Adult cattle	p-value
WH (cm)			
Female	80.3 ± 0.79	104.2 ± 0.32	0.000
Male	91.5 ± 0.15	115.1 ± 0.27	
BL (cm)			
Females	74.5 ± 0.55	101.6 ± 0.13	0.001
Males	88.4 ± 0.74	114.8 ± 0.23	
HG (cm)			
Females	92.8 ± 0.72	132.6 ± 0.25	0.000
Males	107.1 ± 0.13	145.7 ± 0.15	
HL (cm)			
Females	0.91 ± 0.12	7.1 ± 0.32	0.004
Males	1.1 ± 0.21	10.6 ± 0.22	
HC (cm)			
Females	2.18 ± 0.24	10.1 ± 0.42	0.434
Males	2.21 ± 0.31	12.3 ± 0.17	
FL (cm)			
Females	31.3 ± 0.39	42.7 ± 0.31	0.003
Males	39.2 ± 0.52	46.6 ± 0.28	
FW (cm)			
Females	11.16 ± 0.21	14.35 ± 0.11	0.914
Males	13.2 ± 0.34	15.6 ± 0.27	
EL (cm)			
Females	13.4 ± 0.22	16.6 ± 0.24	0.004
Males	17.1 ± 0.32	19.8 ± 0.52	
TL (cm)			
Females	42.20 ± 0.25	68.55 ± 0.72	0.947
Males	52.62 ± 0.92	69.7 ± 0.56	
BW (kg)			
Females	89.6 ± 0.70	180.9 ± 1.12	0.001
Males	110.3 ± 1.32	208.7 ± 1.91	

SE=Standard error, WH=Wither height, BL=Body length, HG=Heart girth, HC=Horn circumference, FL=Face length, FW=Face width, EL=Ear length, TL=Tail length, BW=Body weight

**Figure 2:**
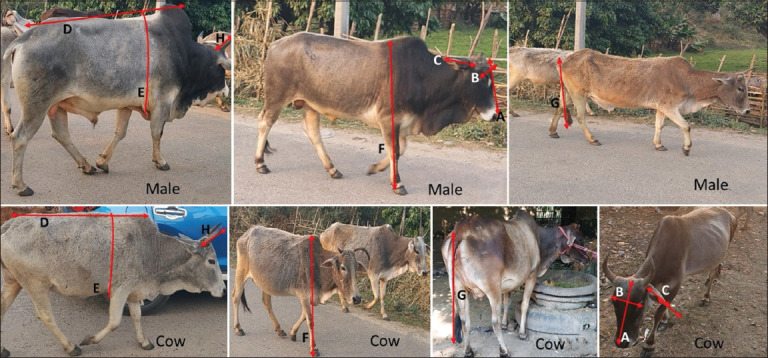
Measurement of (A) head length, (B) Face width, (C) ear length, (D) body length, (E) heart girth, (F) whither height, (G) tail length and, (H) horn length.

The different body measurements (head length [HL], FW, EL, BL, HG, WH, TL, and HL) are shown in Figure 2.

The WH in the females and males were significantly differ (p = 0.000) between the sexes. The BWs of the Tarai cattle were estimated based on Shaeffer’s formula and are given in Table 2. BW also differs significantly (p = 0.001) in females and males. Similarly, the HL, face length (FL), and EL were differ significantly between males and females. On the other hand, HC in females and in males differ (p = 0.434), FW in females and in males differ significantly (p = 0.914). However, no significant differences were found between TL of the females and males.

The cattle of Tarai region were small in stature. The coat color of the animals was predominantly gray to light brown, with white hair on the forehead, neck, and dewlap regions (Figure 2). However, some of the cattle surveyed in the Tarai region exhibited varying degrees of dark brown coat color, especially on the neck and shoulder regions. The forehead was medium in size and flat or slightly convex. The nasal bridge was slightly concave, and the ears were generally erected horizontally. The horns were small to medium in size, generally black in color, and pointed at the tip with curving inward and upward in both males and females. The eyes were commonly small, with black eyelashes. Black muzzle was found in 80% of the Tarai cattle, and pink to dark brown muzzle was found in 20%. In comparison, the legs and neck appear thin and short. In general, the tail and hooves are black.

The hump was comparatively larger in bulls, medium in bullocks, and smaller in size in cows. The udder of the cows was small, and the milk vein was not prominent.

### Production and reproductive performance characteristics

The lactation and reproductive performance traits of Tarai cattle in the North Bihar region are presented in Table 3 The age at sexual maturity, age at first calving, and calving interval were 22.32 ± 0.24 months, 32.44 ± 0.22 months, 16.2 ± 0.12 months, respectively, recorded in Tarai cattle. However, on the other hand, the average milk yield per lactation, peak milk yield, and lactation length were 680.2 ± 4.52 kg, 3.4 ± 0.06 kg, and 224.59 ± 2.06 days, respectively.

**Table 3:** Lactation and reproductive performance traits of Tarai cattle in the northern Bihar region.

Traits	Mean	Range
Milk yield (kg)	680.2 ± 4.52	450–800
Peak milk yield (kg)	3.4 ± 0.06	1.5–4.5
Lactation length (days)	224.59 ± 2.06	200–265
Age at puberty (months)	22.32 ± 0.24	18–27
Age at first calving (months)	32.44 ± 0.22	27–38
Calving interval (months)	16.2 ± 0.12	13–19

## DISCUSSION

### Phenotypic and morphological measurement

The BWs of Tarai cattle were estimated based on Shaeffer’s formula, which is given in Table 2. The adult BW was recorded 180.9 ± 1.12 kg in Tarai cows and 208.7 ± 1.91 kg in Tarai male cattle. Chandran *et al*. [10] reported in Bachaur cattle that the average BW was 246.76 ± 2.42 kg in Bachaur bullocks and 200.55 ± 1.32 kg in Bachaur cows, which is slightly higher than our finding in Tarai cattle. In another study, Dhal *et al*. [11] in Khariar cattle reported mean BWs of 105.40 ± 0.22 kg and 198.22 ± 1.32 kg in adult males and females, respectively, which were lesser than the adult BWs of Tarai cattle found in our study. However, Tarai cattle weighed less than Khillar cattle at different ages [12]. The BWs indicate that Tarai cattle are one of the small breeds of cattle in the North Bihar of *the* Tarai region.

The morphometric parameters of WH, BL, HG, and BW were significantly (p < 0.01) greater in males than in females, except for HC, FW, and TL. Similar morphological characteristics were found in Kedah-Kelantan cattle (*Bos indicus*) in Malaysia, and it was reported that the morphometric parameters of length, width, and circumference were significantly (p < 0.01) greater in Kedah–Kelantan cattle males than in females, except for TL and tail girth [13]. However, when compared with measurements from other indigenous breeds such as Bachaur, Manipuri, Kankrej, Kosali, Ponwar, and Assam, from different states of India (Tables 4 and 5) [10, 14–18], it was observed that these breeds show sex-based differences in their morphological characteristics [11, 14].

**Table 4:** Differences in morphometric measurements between Tarai cattle and various indigenous cows in India.

Breed	Indian state	BL (cm)	HG (cm)	WH (cm)	References
Tarai cattle	North Bihar	101.6 ± 0.13	132.6 ± 0.25	104.2 ± 0.32	Our study
Bachaur	Bihar	113.4 ± 0.4	145.7 ± 0.4	115.9 ± 0.4	[10]
Kosali	Chhattisgarh	96.56 ± 1.87	120.1 ± 2.1	99.79 ± 2.8	[14]
Manipuri local	Manipur	98.92 ± 1.6	133.8 ± 2.7	104.7 ± 1.7	[15]
Kankrej	Gujrat	123.4 ± 0.4	162.6 ± 11.3	124.5 ± 5.6	[16]
Ponwar	Uttar Pradesh	97.1 ± 0.5	140.6 ± 0.5	108.9 ± 0.4	[17]
Assam	Assam	76.14 ± 0.8	104.61 ± 0.9	85.79 ± 0.7	[18]

BL=Body length, HG=Heart girth, WH=Wither height

**Table 5:** Differences in morphometric measurements between Tarai cattle and various indigenous cattle bulls in India.

Breed	Indian state	BL (cm)	HG (cm)	WH (cm)	References
Tarai cattle	North Bihar	114.8 ± 0.23	145.7 ± 0.15	115.1 ± 0.27	Our study
Bachaur	Bihar	113.4 ± 0.4	145.7 ± 0.4	115.9 ± 0.4	[10]
Kosali	Chhattisgarh	96.56 ± 1.87	120.1 ± 2.1	99.79 ± 2.8	[14]
Manipuri	Manipur	98.92 ± 1.6	133.8 ± 2.7	104.7 ± 1.7	[15]
Kankrej	Gujrat	123.4 ± 0.4	162.6 ± 11.3	124.5 ± 5.6	[16]
Ponwar	Uttar Pradesh	97.1 ± 0.5	140.6 ± 0.5	108.9 ± 0.4	[17]
Assam	Assam	76.14 ± 0.8	104.61 ± 0.9	85.79 ± 0.7	[18]

BL=Body length, HG=Heart girth, WH=Wither height

The mean values of the morphometric traits observed in the adult Tarai cattle are comparable to the findings reported by Chandran *et al*. [10] and Singh *et al*. [19] in Bachaur cattle. The breed had comparatively lower morphometric values in Hallikar and Khillar cattle [20]. However, the mean morphometric values of the Tarai cattle found in this study were higher than those reported by Dhal *et al*. [11] in Khariar. These variations in traits among different Indian breeds may be due to differences in genotypes, changes in environmental factors, and interactions between genotype and environment. Morphometric measurements in cattle have been reported earlier and may differ significantly due to factors such as age and sex in various livestock breeds [16]. The results of the present study related to morphometric parameters are comparable to other indigenous cattle of India, particularly of North Bihar breeds, which require more attention to improve these Tarai cattle.

Chandran *et al*. [10] reported that the age at first calving in Bachaur cows was 31.55 ± 0.35 months, and the calving interval was 14.44 ± 0.22 months, which is in close agreement with our finding in Tarai cows. On the other hand, Dhal *et al*. [11] reported a longer age at first calving of 1,540.99 ± 4.29 days and a calving interval of 510.97 ± 2.64 days in Khariar cattle, whereas others reported more or less closer values of calving interval (15 months) in Red Kandhari cows.

The average daily milk yield of cows is 680.2 ± 4.52 kg (range 450–800 kg). However, the peak milk yield was recorded at 3.4 ± 0.06 kg with a range of 1.5–4.5 kg. The cows are milked once or twice a day to meet the requirement of daily family consumption but are generally not for sale. Chandran *et al*. [10] reported in another study of Bachaur cows that the mean lactation yield was 752.10 ± 5.82 kg with an average peak yield of 4.70 kg/day, which is slightly higher than the present study of Tarai cows. However, the average daily milk yield of Purnea cows was reported to be 0.5–2.0 kg, with approximately 4.5% fat [21], which was lower than our finding. Singh *et al*. [19] reported lactation yields and peak yields of 569.13 ± 46.24 L and 3.09 ± 0.24 L, respectively, which were also lesser than the findings of our study.

The mean lactation length in our study was recorded 224.59 ± 2.06 days, varying between 200 and 265 days. The lactation length observed in this study is in close agreement with the lactation length of Bachaur cows, which was 258.79 ± 2.26 days with a range of 220–280 days [10]. In another study, the lactation length was reported to be 230–270 days in Red Kandhari cattle [20], which is more or less consistent with our findings. Overall, the Tarai cows are considered small in body size, and the milk production potential of Tarai cows is lower but cannot be rated inferior as compared with other cattle breeds such as Bachaur and Purnea of Bihar, which is considered as one of the draught purpose cattle breeds of North Bihar.

The non-availability of green fodder and agricultural by-products throughout the year might also explain the lower milk production in Tarai cows. However, the farmers who raised Tarai cattle did not experience many reproductive problems. After drying in the sun, dung from these Tarai cattle was frequently used by farmers as fuel in addition to other agricultural operations like draught power.

## CONCLUSION

It can be concluded that the predominant coat color in Tarai cattle is mostly gray to light brown, with a few other color patterns, a medium-to-small-sized hump, and a dewlap that resembles the local zebu cattle of Bihar. Morphometric parameters of length, width, and circumference were higher in males than in females. This is the first study to use a rigorous scientific methodology to examine the phenotypic and morphometric traits of Tarai cattle in the northern Bihar region.

The findings of this study can be used as a reference for the development and identification of new cattle breeds based on the morphometric and phenotypic traits of North Bihar’s Tarai region. Establishing a long-term selective breeding program in this Tarai region could also benefit from the study. Therefore, additional research to analyze the genetic data may also be conducted to produce a new cow breed exclusive to the Tarai region.

## AUTHORS’ CONTRIBUTIONS

KM: Designed and conducted the study, collected and interpreted data, and wrote the original draft of the manuscript. PK: Data compilation and interpretation, editing, data analysis, and resource person during survey work. AK: Selection of the survey areas, supervision and administration of the project, and reviewed and edited the manuscript. All authors have read and approved the final manuscript.
